# Visual Attentional Bias Induced by Face Direction

**DOI:** 10.3389/fpsyg.2020.01089

**Published:** 2020-05-26

**Authors:** Hui Kou, Nanling Gong, Wenyu Yu, Qinhong Xie, Taiyong Bi

**Affiliations:** ^1^Center for Mental Health Research in School of Management, Zunyi Medical University, Guizhou, China; ^2^School of Criminal Justice, China University of Political Science and Law, Beijing, China

**Keywords:** spatial cueing effect, face direction, eye gaze, SOA, inverted face

## Abstract

The effect of spatial cueing on eye gaze has been confirmed by a large number of studies, but the effect of spatial cueing on face direction and the impact of eye gaze on this effect are less known. In four experiments, we investigated the attentional bias induced by face direction. A modified paradigm of spatial cueing was adopted with stimuli that were static faces rotated by 90 or 45° to the left or right from the frontal view. To control the effect of eyes, face stimuli with eyes open and those with eyes closed were both used in each experiment. In Experiment 1, the facial cue (face rotated by 90°) and target were presented simultaneously, and the stimulus onset asynchrony (SOA) between the facial cue and target was set to be 300, 600, and 900 ms in Experiments 2 (face rotated by 90°), 3 (inverted face rotated by 90°), and 4 (face rotated by 45°), respectively. The response time of detecting the target position was recorded. The spatial cueing effects were nonsignificant in Experiment 1, in which the cue and target were presented simultaneously. However, significant spatial cueing effects of face direction were found in Experiments 2 and 3, in which the upright and inverted faces rotated by 90° were adopted, respectively, in both the eyes open and eyes closed conditions. In addition, we did not find an effect of spatial cueing with the face rotated by 45° (Experiment 4). Our results indicate that face direction can bias visual attention. This effect might not be based on the holistic processing of faces.

## Introduction

The direction of eye gaze is a cue reflecting an individual’s attentional focus or behavioral intentions, and it plays an important role in social interactions. Attentional bias induced by gaze following is widely observed in adult humans (Itier and Batty, 2009) and even infants (Senju and Csibra, 2008; Gredebäck et al., 2010) and animals (Bräuer et al., 2005; Téglás et al., 2012). As it is an endogenous spatial cue, the effect of gaze on attention is usually investigated through a spatial cueing paradigm (Posner and Cohen, 1984). Specifically, a face with an averted gaze is presented as a spatial cue, while a target is presented at the cued (valid cue condition) or the opposite (invalid cue condition) location. Attentional bias is measured by the difference in performance (usually the response time, RT) in identifying the position of the cue between the valid and invalid conditions. The effect of attention on gaze has been found in a variety of studies through the spatial cueing paradigm (Al-Janabi and Finkbeiner, 2014; Manera et al., 2014; Wiese et al., 2014; Cole et al., 2015). Furthermore, the effect of spatial cueing on gaze is also considered automatic and unconscious (Sato et al., 2007), indicating that eye gaze information is processed automatically.

Similar to eye gaze, face direction is also an indicator of one’s attention and plays a similar role as eye gaze in social interactions. However, the results regarding the spatial cueing effects induced by different face directions are inconsistent. On the one hand, some researchers observed a spatial cueing effect induced by different face directions. A study adapting faced rotated by 40° as cues found that whether the eyes were hidden or visible, the participants reacted faster to the targets cued by the robot or human faces than to uncued targets (Chaminade and Okka, 2013). On the other hand, a weak spatial cueing effect or the absence of an effect was found in other studies. For example, Hietanen (1999) found that a profile view (30°) of a face with a compatible eye gaze direction did not result in a spatial cueing effect. It is thus important that we identify the reason for the discrepancy in the results and reconcile the discrepancy between the two kinds of results.

The first factor that may affect the spatial cueing effect of face direction is the visibility of the eyes. Some studies have proposed that face direction alone is not sufficient to induce the spatial cueing effect and that the involvement of gaze cues is necessary. A previous study revealed that observers respond quickly to target locations cued by the direction of a face with eyes open rather than those cued by the direction of a face with closed eyes (Nuku and Bekkering, 2008). It is worth noting that in that study, the results also showed the presence of a spatial cueing effect of face direction when the face had sunglasses but not when the eyes were concealed by occluders (Nuku and Bekkering, 2008). A recent study investigated the effects of face direction and gaze direction in face-to-face communication and revealed that only visible eye gaze (and not visible face direction) can direct an individual’s attention to a target (Hanna et al., 2020). These findings demonstrate that the visibility of eye gaze, even the imagination of gaze information, is crucial in generating attentional bias. In contrast, other studies suggest that eye gaze information may not be necessary in generating the spatial cueing effect of face direction. Some researchers found that regardless of whether the eyes were visible or occluded, evident spatial cueing effects of face direction could be observed (Chaminade and Okka, 2013). Note that in that study, the eyes were rendered occluded by marking the eye regions black. Therefore, the existing evidence does not present consistent information on the role of eye information in generating the spatial cueing effect of face orientation. In the present study, we investigated this effect by adapting face stimuli with eyes open and those with eyes closed and comparing the results between these two conditions.

Second, the spatial cueing effect of face direction might be modulated by the stimulus onset asynchrony (SOA) between a cue and a target. However, the range of SOA in which the cues are effective has still not been determined. A previous study demonstrated that the spatial cueing effect of face direction was evident when the SOA was 100 ms rather than 500 or 1000 ms (Langton and Bruce, 1999). Another study found that the effect was evident with an SOA of 300 ms, regardless of whether the eyes were hidden or open (Chaminade and Okka, 2013). However, at SOAs of 170 and 220 ms, Hietanen (1999) did not find a spatial cueing effect. Furthermore, in other studies, researchers have even observed a reverse effect of cueing at a relatively long SOA, indicating an effect of inhibition of return. For example, a study found a significant spatial cueing effect at SOA values of 200 and 350 ms. This facilitation effect became an inhibition effect at an SOA of 650 ms (Nuku and Bekkering, 2008). Therefore, it is necessary to examine the time course of the spatial cueing effect of face direction. In the present study, we aimed to examine the spatial cueing effect at different stages of face processing. As described above, researcher usually found significant cueing effects at SOAs less than 350 ms. Therefore, the earliest stage in the present study is set at an SOA of 300 ms. Next, as previous findings were not consistent around the SOA of 500∼650 ms, we set the second stage at an SOA of 600 ms. Finally, the latest stage is set at an SOA of 900 ms so that the intervals between each two adjacent SOAs are the same.

Third, few studies have investigated the impact of face inversion on the spatial cueing effect of face direction. Some results indicate that face inversion may largely eliminate the spatial cueing effect of eye gaze (Kingstone et al., 2000). Regarding the spatial cueing effect of face direction, one study found the presence of a spatial cueing effect of face direction in inverted faces, indicating that face inversion has little influence on the cueing effect (Langton and Bruce, 1999). Investigations on the face inversion effect may reveal crucial evidence on the mechanisms underlying the spatial cueing effect of face direction. If face inversion eliminates the cueing effect, the attentional processing of face direction may rely on the holistic information of face stimuli. Therefore, we examined the spatial cueing effect of inverted faces in Experiment 3.

Finally, the impact of deviations in the profile of a face from the frontal view on the spatial cueing effect of face direction remains largely unknown. The gaze cueing effect has been shown to increase with the degree to which the gaze is averted (Qian et al., 2013). However, few studies concerning the spatial cueing effect of face direction have compared the effects among faces rotated by different angles. Previous studies have only showed that the spatial cueing effects are significant when the faces are rotated by approximately 90° (Langton and Bruce, 1999), 40° (Chaminade and Okka, 2013), and 15° (Nuku and Bekkering, 2008). However, it should be intuitive that the less a face is rotated, the smaller the cueing effect. Therefore, in the present study, we used faces that were rotated by a moderate angle of 45° in Experiment 4 and faces rotated by an extreme angle of 90° in Experiments 1, 2, and 3 to compare the spatial cueing effects between the two kinds of faces.

In summary, we aimed to investigate the spatial cueing effect of face direction and its influencing factors. In Experiment 1, faces that were rotated by 90° were used as the cue, and the target was presented simultaneously with the cue. In Experiment 2, three SOAs between the cue and the target were set to investigate the time course of the spatial cueing effect of face direction. In Experiment 3, inverted faces rotated by 90° were used to examine the spatial cueing effect with inverted faces. In Experiment 4, faces rotated by 45° were used to examine whether the spatial cueing effect becomes more evident when the faces are rotated away from the frontal view. In the last three experiments, face cues were presented for 200 ms, which was shown long enough to induce significant cueing effect in previous studies (Langton and Bruce, 1999; Nuku and Bekkering, 2008).

## Experiment 1: The Cue and Target Were Presented Simultaneously

### Participants

A total of 30 naïve subjects (18 females and 12 males) participated in this study. They were right-handed with reported normal or corrected-to-normal vision. All of them were college students (*M* = 20.60 years, SD = 1.28, age-range = 18–23). No histories of neurological or psychiatric problems were reported. They signed written informed consent forms that were approved by the Institutional Human Participants Review Board of Zunyi Medical University in China. The study was conducted in accordance with the Declaration of Helsinki.

### Materials

A three-dimensional (3D) face model was generated by FaceGen Modeller 3.1^1^. No hair was rendered. The face model was the default average face in the software, and the value of texture gamma correction was set to 2. This model was used in all the experiments.

**FIGURE 1:**
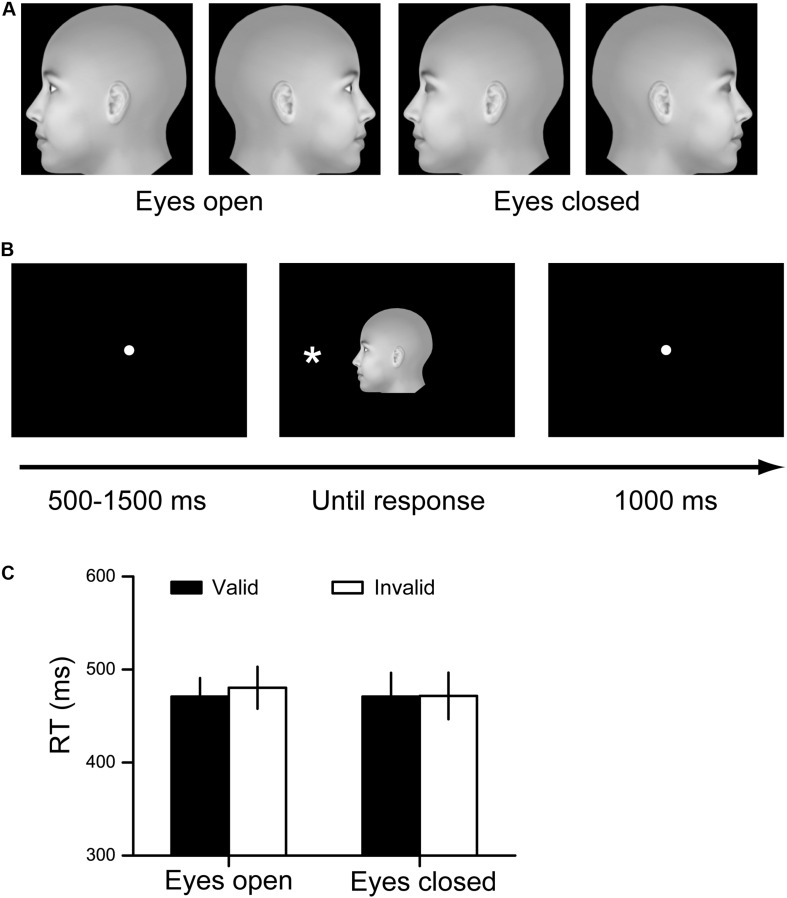
Experimental procedure and results for Experiment 1. **(A)** Stimuli used in Experiment 1. **(B)** The paradigm of spatial cueing. The facial cue and the target were presented simultaneously. The cue is valid when the face orients toward the target. Otherwise, the cue is invalid. **(C)** The RT results for the eyes open condition and the eyes closed condition. Black bars denote the average RTs to the valid cue, while white bars denote the average RTs to the invalid cue. Data were averaged across all subjects. Error bars denote one standard error of the mean.

The visual stimuli for cueing were generated by projecting a 3D stimulus model with different in-depth rotation angles onto the monitor plane with the front view (0°) as the initial position. The rotation angles were −90° to generate the left profile face and 90° to generate the right profile face. In addition, all faces were generated with either the eyes open or closed. Therefore, four face stimuli were finally generated: a left profile face with eyes open, a right profile face with eyes open, a left profile face with eyes closed and a right profile face with eyes closed (Figure 1A). The stimuli extended to 8° × 8° of the visual angle. The stimulus of the target was an asterisk (^∗^) with a 0.8° × 0.8° visual angle, and the stimulus could be presented on either the left side or the right side of the point of central fixation. The angular distance between the target and the point of fixation was 7.8°. The visual stimuli were presented on an SAMSUMG 19-in LCD screen, with a spatial resolution of 1280 × 800 and a refresh rate of 60 Hz (Zhang et al., 2018). The subjects viewed the stimuli from a distance of 60 cm. Throughout the experiments, the subjects were asked to fixate on a small white dot that appeared in the center of the monitor.

### Procedure

We utilized a modified paradigm of spatial cueing in this study. At the beginning of each trial, a white fixation point was displayed in the center of the black screen for a random period of 500∼1500 ms. Subsequently, one of the face stimuli was presented in the center of the screen, and an asterisk was simultaneously displayed on the left or right side of the face. The participants were asked to press one key (F) if the asterisk was presented on the left side and to press another key (J) if the asterisk was presented on the right side, as quickly and accurately as possible. The stimuli disappeared after the response was recorded, and a 1000 ms blank screen was then presented (Figure 1B). In this experiment, each participant completed four testing blocks. In two of the blocks, the eyes on the faces were open, and in the other two blocks, the eyes of the faces were closed. The order of blocks was random for each participant. Each block consisted of 48 trials. The face directions and target locations were counterbalanced within each block, resulting in a 50% cue validity for each condition.

### Designs

The experimental design was a 2 (cue validity: valid/invalid) × 2 (eye visibility: open/closed) factorial study design. Both cue validity and eye visibility were included in the analysis as within-subject factors.

### Data Analysis

The accuracy was first calculated for each condition and each participant. The reaction time (RT) was then calculated by excluding the trials with incorrect responses. Repeated measures ANOVA was conducted on the accuracy and RT results. *Post hoc* analysis with Bonferroni adjustments was conducted when a main effect or interaction effect was found to be significant.

### Results

The accuracy was quite high (>99%) for each condition. Repeated measures ANOVA showed that no interaction or main effects of the study factors on accuracy were significant.

The RT results are illustrated in Figure 1C. We did not find any significant interaction or main effects for the RT results (all *F* < 1, *p* > 0.4, η^2^ < 0.023).

## Experiment 2: The Cue and Target Were Presented Sequentially

The results from Experiment 1 showed no spatial cueing effects of face direction when the cue and target were presented simultaneously. To further investigate the time course of the cueing effect, the cue and target were presented sequentially in this experiment.

### Participants

A total of 30 naïve subjects (16 females and 14 males) participated in this study. They were right-handed with reported normal or corrected-to-normal vision. All of them were college students (*M* = 20.30 years, SD = 1.18, age-range = 18–24). No histories of neurological or psychiatric problems were reported. They signed written informed consent forms that were approved by the Institutional Human Participants Review Board of Zunyi Medical University in China. The study was conducted in accordance with the Declaration of Helsinki.

### Materials

All the materials were the same as those used in Experiment 1.

### Procedure

**FIGURE 2:**
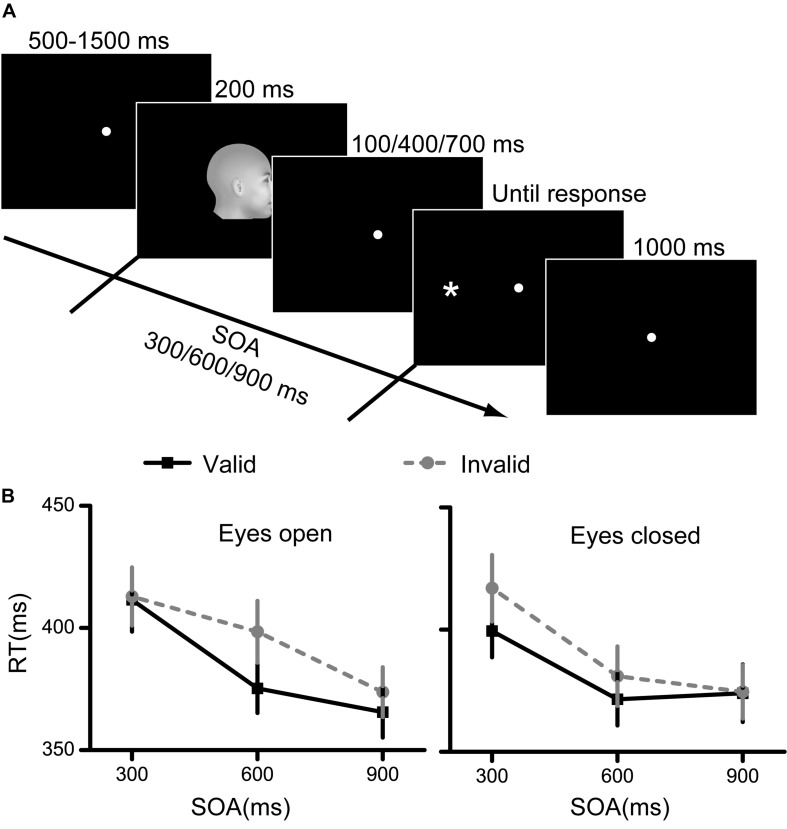
Experimental procedure and results for Experiment 2. **(A)** The paradigm of spatial cueing used in Experiment 2. The facial cue and the target were presented sequentially. The facial cue was presented for 200 ms and the stimulus onset asynchrony between the cue and target were 300, 600, or 900 ms. **(B)** The RT results for the eyes open condition and the eyes closed condition. Black squares denote the average RTs to the valid cue, while grey circles denote the average RTs to the invalid cue. Data were averaged across all subjects. Error bars denote one standard error of the mean.

The procedure was similar to that in Experiment 1 (Figure 2A). In each trial, a blank screen with fixation was first presented for 500∼1500 ms. Afterward, a facial cue was presented for 200 ms, followed by a blank screen that was presented for 100, 400, or 700 ms. The target was presented immediately after the blank interval. The SOA between the facial cue and target was defined as the time interval between the onset times of the face and target. Therefore, the SOAs included in the present experiment were 300, 600, and 900 ms. The participants were also asked to indicate the position of the target as quickly and accurately as possible. In this experiment, each participant completed 12 testing blocks. In six blocks, the eyes of the faces were open, and in the other six blocks, the eyes of the faces were closed. Each block consisted of 48 trials. The face directions, target positions and SOAs were counterbalanced in each block.

### Designs

The experimental design is a 2 (eye visibility: open/closed) × 2 (cue validity: valid/invalid) × 3 (SOA: 300/600/900 ms) factorial design. All the independent variables were included in the analysis as within-subject variables.

### Data Analysis

The procedure used to analyze the data was the same as that used in Experiment 1.

### Results

The accuracy was quite high (>98%) for each condition. Repeated measures ANOVA showed that no interaction or main effects were significant for the accuracy results.

The RT results are illustrated in Figure 2B. First, the interaction among the three factors was significant [*F*(2,58) = 3.832, *p* = 0.027, η^2^ = 0.117]. Second, the interaction between eye visibility and SOA was significant [*F*(2,58) = 3.633, *p* = 0.033, η^2^ = 0.111]. Finally, the main effects of cue validity and SOA were both significant [cue validity: *F*(1,29) = 18.752, *p* < 0.001, η^2^ = 0.393; SOA: *F*(2,58) = 54.181, *p* < 0.001, η^2^ = 0.651]. The results of the main effect of cue validity indicated a significant cueing effect of face direction. However, the interaction results indicated that the cueing effect may be different for faces with eyes open and closed. Therefore, we separately analyzed the results for the eyes open and eyes closed conditions.

Two 2 (cue validity) × 3 (SOA) repeated measures ANOVA models were used for the RT results for faces with eyes open and closed.

When the eyes were open, no significant interaction effect was found between cue validity and SOA. However, the main effects of cue validity and SOA were both significant [cue validity: *F*(1,29) = 16.991, *p* < 0.001, η^2^ = 0.369; SOA: *F*(2,58) = 34.078, *p* < 0.001, η^2^ = 0.540]. The *post hoc* tests showed that the RT decreased with the SOA (all *p* < 0.05 for the comparisons among the three SOAs).

When the eyes were closed, the results showed a significant interaction effect between cue validity and SOA [*F*(1,29) = 3.694, *p* = 0.031, η^2^ = 0.113], as well as significant main effects of both factors [cue validity: *F*(1,29) = 5.819, *p* = 0.022, η^2^ = 0.167; SOA: *F*(2,58) = 39.447, *p* < 0.001, η^2^ = 0.576]. These results indicated different cueing effects among different SOAs.

To examine the spatial cueing effects more straightforward, we further conducted direct comparisons between valid and invalid conditions at each SOA and each eye visibility condition. Results showed a significant spatial cueing effect (lower RT for the valid condition compared with the invalid condition) only at an SOA of 600 ms [paired *t-*test, *t*(29) = −3.466, *p* = 0.006, Bonferroni corrected] when the eyes are open; and showed a significant spatial cueing effect at an SOA of 600 ms [paired *t*(29) = −2.834, *p* = 0.024, Bonferroni corrected], and a marginally significant spatial cueing effect at an SOA of 300 ms [paired *t*(29) = −2.372, *p* = 0.075, Bonferroni corrected], when the eyes are closed. Other spatial cueing effects were nonsignificant.

Taken together, the results from Experiment 2 demonstrated that the spatial cueing effects of face direction were evident regardless of whether the eyes were open or closed. However, the cueing effects might be most evident at an SOA of 600 ms whether the eyes were open or closed.

## Experiment 3: Spatial Cueing Effect of Inverted Faces

To explore whether the spatial cueing effect is induced by local features or holistic information regarding the face, we turned the faces upside down and measured the spatial cueing effect of these inverted faces.

### Participants

A total of 30 naïve subjects (23 females and 7 males) participated in this study. They were right-handed with reported normal or corrected-to-normal vision. All of them were college students (*M* = 20.33 years, SD = 1.42, age-range = 18–23). No histories of neurological or psychiatric problems were reported. They signed written informed consent forms that were approved by the Institutional Human Participants Review Board of Zunyi Medical University in China. The study was conducted in accordance with the Declaration of Helsinki.

### Materials

**FIGURE 3:**
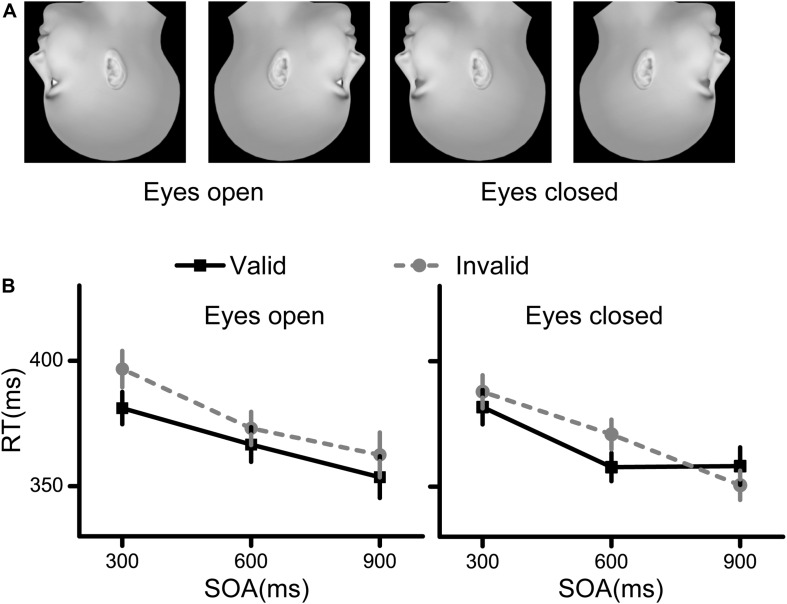
Stimuli and results for Experiment 3. **(A)** The stimuli used in Experiment 3 were inverted faces. **(B)** The RT results for the eyes open condition and the eyes closed condition. Black squares denote the average RTs to the valid cue, while grey circles denote the average RTs to the invalid cue. Data were averaged across all subjects. Error bars denote one standard error of the mean.

The procedure used to generate the face stimuli was the same as that used in Experiment 1. We then turned all the face stimuli upside down (Figure 3A).

### Procedure

The procedure was the same as that used in Experiment 2.

### Design

The experimental design is a 2 (eye visibility: open/closed) × 2 (cue validity: valid/invalid) × 3 (SOA: 300/600/900 ms) design. All the independent variables were included in the analysis as within-subject variables.

### Data Analysis

The procedure used to analyze the data was the same as that used in Experiment 1.

### Results

The accuracy was quite high (>98%) for each condition. Repeated measures ANOVA showed that no interaction or main effects were significant for the accuracy results.

The RT results are illustrated in Figure 3B. First, there was a significant interaction effect between cue validity and SOA [*F*(2,58) = 3.235, *p* = 0.047, η^2^ = 0.100], indicating that there were different cueing effects among the different SOAs. Second, the main effects of cue validity and SOA were both significant [cue validity: *F*(1,29) = 13.313, *p* = 0.001, η^2^ = 0.315; SOA: *F*(2,58) = 41.420, *p* < 0.001, η^2^ = 0.588). The other interaction and main effects were not significant (all *p* > 0.05).

Next, simple effect analysis was conducted corresponding to the significant interaction effect. Planned comparisons between the valid and invalid conditions showed significant spatial cueing effect at SOAs of 300 ms (*p* = 0.024, Bonferroni corrected) and 600 ms (*p* = 0.003, Bonferroni corrected), but not at an SOA of 900 ms (*p* > 0.05).

The results from Experiment 3 indicated that the spatial cueing effects of face direction in inverted faces were significant at SOAs of 300 and 600 ms, regardless of the visibility of eyes.

## Experiment 4: The Spatial Cueing Effect of a Face Rotated by 45°

The results above showed a significant cueing effect for faces rotated by 90° under certain circumstances. To further investigate whether this effect is also evident in faces rotated by smaller degrees, we used faces rotated by 45° from the frontal view.

### Participants

A total of 30 naïve subjects (22 females and 8 males) participated in this study. They were right-handed with reported normal or corrected-to-normal vision. All of them were college students (*M* = 20.30 years, SD = 1.49, age-range = 18–23). No histories of neurological or psychiatric problems was reported. They signed written informed consent forms that were approved by the Institutional Human Participants Review Board of Zunyi Medical University in China. The study was conducted in accordance with the Declaration of Helsinki.

### Materials

**FIGURE 4:**
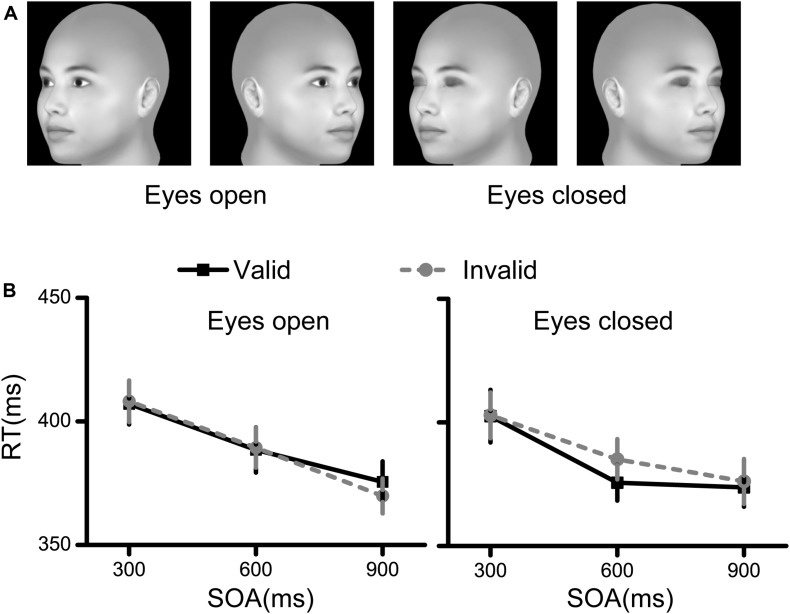
Stimuli and results for Experiment 4. **(A)** The stimuli used in Experiment 4 were faces rotated by 45°. **(B)** The RT results for the eyes open condition and the eyes closed condition. Black squares denote the average RTs to the valid cue, while grey circles denote the average RTs to the invalid cue. Data were averaged across all subjects. Error bars denote one standard error of the mean.

The procedure used to generate the face stimuli was the same as that used in Experiment 1. When we rotated the faces, the rotation angles were set to −45° to generate the left profile face and 45° to generate the right profile face (Figure 4A).

### Procedure

The procedure was the same as that used in Experiment 2.

### Design

The experimental design is a 2 (eye visibility: open/closed) × 2 (cue validity: valid/invalid) × 3 (SOA: 300/600/900 ms) design. All the independent variables were included in the analysis as within-subject variables.

### Data Analysis

The procedure used to analyze the data was the same as that used in Experiment 1.

### Results

The accuracy was quite high (>99%) for each condition. Repeated measures ANOVA showed that no interaction or main effects were significant for the accuracy results.

The RT results are illustrated in Figure 4B. Only the main effect of SOA was significant [*F*(2,58) = 43.017, *p* < 0.001, η^2^ = 0.597], while the other main effects and interaction effects were nonsignificant (all *p* > 0.05, η^2^ < 0.11).

These results demonstrated no spatial cueing effect of faces rotated by 45°.

## Discussion

In the present study, we explored the spatial cueing effect of face direction and its impact factors. We found that regardless of whether the eyes were open or closed, the spatial cueing effects of the faces rotated by 90° were observed at certain SOAs. Such cueing effects were also found for inverted faces, indicating that the spatial cueing effect might be mainly affected by local rather than holistic information on faces. In addition, these effects disappeared for faces that were rotated by 45°. These findings contribute significantly to the understanding about the attentional processing of face direction. First, the present study confirmed the attentional bias induced by face direction, which is not consistently found in previous studies. Second, more importantly, our results provide critical pieces of evidence on the mechanism of such attentional processing of face direction. Local features but not holistic processing are implied to play a crucial role in generating the attentional bias. Furthermore, eye visibility is shown to have little impact on the cueing effect of face direction, which indicates that the processing of face direction may be independent of the processing of gaze. Third, the investigations on the effective SOA and rotation angle of face direction may help reconcile the discrepancy in previous literatures revealing inconsistent cueing effects. Our results indicate that the attentional bias could be generated only in a specific range of SOA and in a specific range of rotation angle, which might explain why some studies found evident cueing effect while others did not.

We found that regardless of whether the eyes were open or closed, the spatial cueing effects of face direction were observed at certain SOAs when the faces were rotated by 90° from the frontal view, indicating that eye visibility had a limited impact on the cueing effect of face direction. This result implies that the attentional processing of face direction might not be based on information provided by the eyes on faces. Our results were consistent with previous findings, which also showed little influence of eye visibility on the cueing effect of face direction (Chaminade and Okka, 2013). At the neural level, although there is little direct evidence showing the effect of eye visibility on face direction processing, the existing fMRI evidence indicates that the processing of face direction may not be influenced by eye-related information. For example, the neural responses to front-view faces were shown to be higher in both the fusiform gyrus (FG) and posterior superior temporal sulcus (pSTS) than those to profile faces, regardless of the gaze direction (Pageler et al., 2003). As eye gaze processing has been well investigated, our results demonstrated that the processing of face direction may not be identical to that of eye gaze and requires further investigation.

In the present study, we did not find a significant interaction between SOA and cue validity from the results of the eyes open condition, indicating that the spatial cueing effect did not change with the SOA. However, it should be noted that, when examined the cueing effect directly, the spatial cueing effect of face direction with eyes open was only significant at an SOA of 600 ms. Although further studies are needed to draw a more solid conclusion, our results might indicate that at least there is a trend that the cueing effect is most evident around 600 ms SOA, regardless of the visibility of eyes. It seems inconsistent with previous studies that showed a significant spatial cueing effect of face direction at more earlier SOAs of 100–350 ms but not at 500–650 ms (Langton and Bruce, 1999; Nuku and Bekkering, 2008). One possible reason for the discrepant findings between the present and previous studies might be the way of cue presentation. In the present study, the cue was always presented for 200 ms, which resulted in a gap between the disappearance of the cue and the appearance of the target. When the cue disappeared, the precision of attention may decrease with time. When the target appeared, it may take time for attention to relocate to the area of target presentation. However, there was no gap between the cue and the target in previous studies, saving the time for attention relocation. Nevertheless, what is more important, both the present and the previous results consistently demonstrated that there was a specific time window in which the attentional effect of face direction was effective. However, the range of the time window needs to be validated further with more studies.

An especially interesting finding in our study might be the evident spatial cueing effect of inverted faces. It has been proposed that there are distinct processing mechanisms used for inverted and upright faces (Yin, 1969). The most important difference between them is holistic processing, which is fundamental for upright faces but not used for inverted faces. Neuroimaging evidence has also revealed the involvement of different neural substrates for processing upright and inverted faces in the fusiform face area (FFA) (Yovel and Kanwisher, 2005). Face inversion may disrupt the configural processing and holistic representation of faces. Therefore, the processing that is dependent on holistic information on a face can also be influenced by face inversion. The present study demonstrated that the spatial cueing effect of face direction was not affected by face inversion, which was consistent with the findings in a previous study that revealed a spatial cueing effect of face direction in inverted faces (Langton and Bruce, 1999). However, it should be noted that, the spatial cueing effect of inverted face was only marginally significant in the previous study. Therefore, the conclusion is not convincing. The present study revealed a more stable cueing effect, and thus confirmed that the attentional processing of face direction might depend on local features of the face rather than holistic information. The specific local features that are crucial for the cueing effect of face direction require further investigation. Additionally, the present study also revealed that the spatial cueing effects were significant at SOAs of 300 and 600 ms, which was not examined in previous studies. Compared with the results of Experiments 2 and 3 found a more significant cueing effect at an SOA of 300 ms, which was the main difference between the two experiments. This difference reminded us that face inversion may have a certain impact on the attentional processing of face direction. One possible reason might be that, according to the face inversion effect, subjects may process local features better in inverted faces rather than in upright faces. Therefore, the present findings may strongly support that the attentional bias induced by face direction largely depends on the processing of local features in the face.

Finally, the present study did not reveal a significant spatial cueing effect of face direction with faces rotated by 45° from the frontal view. This result further supports the idea that the facial local features might be crucial factors for the spatial cueing effect of face direction because the cues of the directions were much less prominent than the rotation of the faces by 90°. However, a few previous studies observed a significant spatial cueing effect of face direction with faces that were rotated by smaller degrees. For example, the study conducted by Chaminade and Okka (2013) used faces that were rotated by 40°; the study conducted by Nuku and Bekkering (2008) adopted faces that were rotated by 15°. These discrepancies between these studies and our study may result in the different findings. For example, the duration of face presentation was longer in previous studies than in this study. Moreover, the facial cue was presented for 300 ms in the study by Chaminade and Okka (2013); the facial cue was presented until a response was recorded in the study by Nuku and Bekkering (2008). Under such a condition, subjects may extract and process the local feature cues more effectively. However, the effect of the presentation time of facial cues requires further investigation.

There are several limitations in the present study, and additional studies need to be conducted to address them. First, the spatial cueing effect was not observed at the SOA of 0 ms (Experiment 1) but was observed at the SOA of 300 ms (Experiment 2). It is thus necessary to examine the cueing effect during the period of 0 ∼ 300 ms. In addition, it is also worth investigating the range of SOA during which an inhibition effect of the facial cue can be observed. Second, the different results between Experiments 1 and 2 may result from the different presentation durations of the faces between these two experiments. Therefore, further studies are needed to examine how the presentation duration of face cue affects the spatial cueing effect. Third, and most importantly, additional studies are needed to examine the specific facial features that are critical in generating the cueing effect of face direction. Such investigations may help us gain a deeper understanding of the mechanisms of facial direction processing. Finally, it is also interesting to study the effects of other social cues (e.g., eye gaze, facial expression, etc.) on the spatial cueing effect of face direction.

## Conclusion

Significant spatial cueing effects of face direction were observed at certain SOAs, indicating that attentional bias was induced by face direction. However, this attentional effect of face direction may not be based on the processing of holistic facial information.

## Data Availability Statement

The datasets generated for this study are available on request to the corresponding author.

## Ethics Statement

The studies involving human participants were reviewed and approved by the Institutional Human Participants Review Board of Zunyi Medical University. The patients/participants provided their written informed consent to participate in this study.

## Author Contributions

HK and TB contributed conception and design of the study. HK, NG, and WY collected the data. HK and QX performed the statistical analysis. HK and NG wrote the first draft of the manuscript. All authors contributed to manuscript revision, read and approved the submitted version.

## Conflict of Interest

The authors declare that the research was conducted in the absence of any commercial or financial relationships that could be construed as a potential conflict of interest.

## Footnotes

1 http://www.facegen.com/
